# Supplementary material for: Chromothripsis during telomere crisis is independent of NHEJ, and consistent with a replicative origin
Source: Genome Res. 2019 May;29(5):737–49. doi: 10.1101/gr.240705.118 (PMC6499312; doi:10.1101/gr.240705.118)
Supplement: Supplemental Material [file supp_gr.240705.118_Supplemental_file_1.zip › contigs/annotated_contigs/DB53/contig.2.DB53_length_406_mean_cov_5.68965517241.docx]

**DB53_length_406_mean_cov_5.68965517241**

```
TTCCTAGCTCTGAACTGGGGCCCTGGCTGGGCCTAGGACAGACACTGAGTT|TTA|TGCTTTCTTGACATAAAAATAAGGACTTAAAGA
 >chr8:135539774-135539825 - E=4e-19                   >chr8:135550846-135551171 - E=4e
ATTCCAGCTTTTAACACATTCAATCTCATTTTCTCTCAACTCAATTTGTAAATCTTCAATGTACGTCTTTTTAATCTTTATCTTTTTTT
-168
TTTTTTTTTGTGACAGGGTCTCACTTTGTCACCCAGGCTGGAATGCAGTGGCGCAATCTTGGCTCACTGTGACCTCTGTCTCCCAGGTT

CAAGCAATTCTCGTGCCTCAACCCCACAAGTAGCTGGGACTACAGGCATGTACCACCATGCCCGGCTAATTTTTGTATTTTTAGTAGAG

AAGGGGTTTCGCCATGTTACCCAG|AGGACAAAGGAAGGTGGCTTGCAGAGGG
```
